# On Blistering and Topical Cold

**Published:** 1817-08

**Authors:** 


*For the London Medical and Physical Journal.*

### *On Blistering and Topical Cold*; By Dr. KINGLAKE.

THE result of much and close attention to the influence of temperature in the diseased actions of the animal economy, has convinced me that some of the popular notions of appropriate remedies should be dispassionately examined and re-judged. No one can rely with more gratifying confidence than myself on the unequivocally established efficacy of accredited means of cure. It is a most consoling relief under an earnest solicitude to benefit morbid suffering, to be able promptly to resort to an adequate remedy. No speculations implying doubt should be obtruded on such medicinal influence as is unquestionably salutary. In such instances, the direct practical advantages amply counterbalance and set at nought all hypothetical refinements that might be indulged; but, when less certainty exists, when obscurity veils the reputed beneficial power of medicinal agents, it is allowable, nay, laudable, to endeavour to remove all ambiguity, by ascertaining what may be true in the circumstances stated.

Having premised these observations, it may be presumed, that it will not be deemed too adventurous to offer a few remarks on the prevailing practice of blistering the regions of visceral cavities, for the purpose of mitigating or transferring to to the surface the active violence of internal inflammation. The numerous instances in which vesication is employed, on the principle of external derivation from an internal part, afford endless examples of recoveries as well as failures, so that the event of the treatment cannot be justly regarded as decisive on the point in question. Most of the instances would, probably, have recovered without blistering, as they are found to do with it; its influence, therefore, is too doubtful to be admitted as determining the important consideration of life and death.

The familiarity with which the mode of remedy is resorted to, silences all apprehension as to its being injurious; it is held to be at least innoxious, if not likely to effect the desired relief. Regulating my judgment less by relative than by assiduous observation, it has occurred to me to remark, in various instances, that vesication over the cavities in inflammatory affections of the subjacent viscera, has at once greatly aggravated the local disease, and the general irritation usually on these occasions pervading the system. The action of the heart and arteries has been quickened and hardened by its stimulating agency, and, consequently, every morbid condition and feeling have been exasperated. Much attention, however, is requisite to mark the states of vital power in which vesication may either injure or ameliorate morbid action. Where unnatural torpor and deficient re-action exist in diseases, the stimulating effect of blistering may usefully excite the languid and drooping state of life; but, on the contrary, where excessive irritability and violent excitement present, the additional stimulus imparted by vesication would appear to be contra-indicated and necessarily hurtful. The grand distinction to be made in the management of disease is, whether it be founded in excessive or deficient exertion of vital power, because the curative treatment should be adopted accordingly. It will not be contended, that the diseases, or the morbid conditions of the former class, can require further excitement, nor that those of the latter can need additional reduction. Inflammatory diseases then of every description, as well as those of high febrile action, must be founded on immoderate excitement; and, it is in those instances that it would appear, that indiscriminate vesication is seriously injurious. It has lately happened to me, to have seen different cases in which this point has been illustrated, in a way that has gone far to satisfy my judgment on the subject.

In a case of phrenitis, in which bleeding carried to a considerable length had not much availed, and in which shaving and blistering the vertical part of the head, and also the nape of of the neck, rendered the patient wholly unmanageable; the utmost constraint was necessary to confine the struggling sufferer to the bed. This difficulty had been so manifestly increased by the pain induced by the blisters, and the pulse had been so much accelerated by the same cause, that it was thought right to remove them, and to substitute the application of cold water, by means of large towels wetted in that fluid, and enveloping the whole head. With this change of treatment, the delirious ravings soon ceased, and were succeeded by composure and sleep. The intervals of repose kept pace for some hours with the refrigerant influence of the cooling application. When the cloths had nearly acquired the temperature of the scalp by too long a continuance on the part, muttering restlessness re-commenced, which was restrained and silenced by renewing the wet cloths. This treatment, I understood, was unremittedly persisted in during upwards of twelve hours, when the patient became rational, complained only of weakness, and was desirous that cold should be applied to the head if any degree of pain recurred. Convalescence immediately took place, and perfect recovery speedily ensued. Judging from former experience, it appeared to me, that the chance of a favorable termination in this case was too small to be reasonably calculated on; the recovery, therefore, was, in my opinion, justly attributable to the salutary efficacy of topical cold over the region of the brain.

A case lately occurred in my practice of mental derangement of the vehement description, in which the disordered ravings of the mind were shown in violent paroxysms at longer or shorter intervals, according to the influence of accidental circumstances, such as an abrupt admission of light into the patient's apartment, casual sounds, occasional coercion, &c. These paroxysms wasted their force by long continuance, enduring for two or three hours, and then subsiding into a state of calmness and languor approaching to *deliquium animi*. Ample bleeding from the temporal artery, copious discharges from the intestines, and extensive blistering of the scalp, were employed. The morbid excitement, however, proceeded; nor did it abate until the blister was removed from the head, and a cold lotion was applied to the forehead, temples, &c. in the most free manner, renewing it as often as the cloths wetted in the fluid became at all warm. Composure, ending in sound sleep, succeeded its first application, and the occasional employ of it has been always found beneficial in restraining and keeping under the immoderate excitement that otherwise would probably have risen to paroxysmal violence. It is not supposed, that in this this case positive inflammation had been induced on the brain, it was most likely a distempered state of the sensorial or nervous power that proved an inordinate stimulus to the whole system, and the indication of cure consisted in reducing the excessive action by sedative means, and that effect seems to be produced most powerfully by the influence of topical cold.

An instance of peritoneal inflammation, in a middle aged female patient, has fallen under my observation, in which the abdomen was more prominently enlarged than is usual, either in an advanced stage of pregnancy, or in that of ascital dropsy. The tumefaction was insufferably painful on pressure; the pulse was small, quick, and hard; the general strength appeared to be giving way, and there was every prospect of approaching gangrene. The medical practitioner who was in attendance, informed me, that but little more had been done than moving the bowels and fomenting with warm water the swollen abdomen. As no benefit had been afforded by warmth, it was proposed to see what might be effected by cold. Cloths wetted in cold vinegar-and-water were incessantly applied over the whole surface of the distended belly, at short intervals, during some hours, with all the relief that could be desired, and much more than could have been reasonably expected. The patient speedily recovered, and declared that the bulk and pain of the abdomen almost immediately diminished, and soon altogether subsided, after the application of the cloths wetted in the cold fluid.

This is a singularly strong case in proof of the efficacy of topical cold in visceral inflammation. Indeed, no description can convey an adequate idea of the prompt and complete relief derived on this occasion from the refrigerent remedy. There could have been no deception in it. The cause and effect were too closely and evidently conjoined to be either mistaken or at all doubtful. It is difficult to prosecute similar trials in the existing popular prejudices against cooling applications. As if relief were solely to be obtained from violent means, the physiology of the day authorises a reference to vesication on a principle, perhaps, more specious and familiar, than true and salutary. Further experience can alone decide the practical value of the treatment here stated on the authority of a single case.

In a case of painful palpitation at the heart inducing a suspicion of some organic affection of that viscus, in which repeated bleedings and vesication had not succeeded in producing any permanent relief, it was suggested to try the effect of topical cold over the region of the affected part. Much benefit speedily resulted from this remedy. The inordinate action of the heart was lessened, and with it also the attending pain, in a degree far exceeding my expectations.

In enlargements of the knee-joint, called white swellings, when accompanied with pain, topical cold more effectually overcomes the disorganizing irritation existing in the ligamentous and cartilaginous structure, than either vesication or any other stimulating influence. It would be in my power to recite other instances of the beneficial effects of topical cold in reducing inflammatory and distempered irritation; but what has been stated will suffice to justify the principle of endeavouring rather to allay than to excite action in such conditions of disease as are founded in an immoderate exertion of vital power.

By a beneficent providence of nature, happily designed for alleviating the severity of incidental diseases in the animal economy, all unnatural actions of vital power, whether excessive or deficient, have a tendency to cure themselves. Those of the former class, by exhaustion; of the latter, by restoration. Thus, febrile and inflammatory diseases naturally conduce to their own extinction, by dissipating the excessive power on which they mainly depend, whilst the torpid condition of living power derives, from the nutritive and plastic processes of life, recruited energy by diminished action and comparative rest. It is, therefore, obvious, that the one description of diseases requires to be repressed and brought within the sphere of healthy action; and the other to be raised by nutritive and other congenial stimulants, to the standard of health. How to accomplish these salutary objects in the most direct and efficient manner, is the grand medical desideratum. A leading consideration in the management of disease is, so to moderate and regulate the occurring symptoms as to prevent disorganization from happening during the morbid process. If no organic mischief be induced, the fluctuating conditions of living power, whether in excess or deficiency, may be adjusted and directed in a way that may lead to the ultimate re-establishment of health. When the principle of medical practice is settled, the mode of fulfilling the curative indication will legitimately flow from it, and obviously suggest itself to the practitioner. Is too high a degree of excitement, from whatever cause, disordering the action of vital power, let it be suitably diminished; is excitement at too low an ebb, let it be gradually raised to the healthy standard. The outline of the means for effecting these salutary intentions, is, probably, in heat and cold, or in appropriate degrees of temperature.

*Taunton; May* 12, 1817.

