# Erratum

**Published:** 1817-09

**Authors:** 


Erratum.—In page 103, line 14, for *relative*, read *routine*.

